# Supplementary material for: Parvifloron D from Plectranthus strigosus: Cytotoxicity Screening of Plectranthus spp. Extracts
Source: Biomolecules. 2019 Oct 17;9(10):616. doi: 10.3390/biom9100616 (PMC6843537; doi:10.3390/biom9100616)
Supplement: Supplementary file 1 [file biomolecules-09-00616-s001.pdf]

Supplementary Materials

Parvifloron D from *Plectranthus strigosus*: Cytotoxicity Screening of *Plectranthus* spp. Extracts

Catarina Garcia [1,2], Epolle Ntungwe [1,2], Ana Rebelo [1,2], Cláudia Bessa [3], Tijana Stankovic [4], Jelena Dinic [4], Ana Díaz-Lanza [2], Catarina P. Reis [5], Amílcar Roberto [1], Paula Pereira [1,6], Maria-João Cebola [1,6], Lucília Saraiva [3], Milica Pesic [4], Noélia Duarte [5,*] and Patrícia Rijo [1,5*]

[1] Research Center for Biosciences & Health Technologies (CBIOS), Universidade Lusófona de Humanidades e Tecnologias, 1749-024 Lisboa, Portugal
[2] Department of Biomedical Sciences, Faculty of Pharmacy, University of Alcalá, Campus Universitario, 28871 Alcalá de Henares
[3] LAQV/REQUIMTE, Laboratório de Microbiologia, Departamento de Ciências Biológicas, Faculdade de Farmácia, Universidade do Porto, Rua de Jorge Viterbo Ferreira n. 228, 4050-313, Porto, Portugal
[4] Institute for Biological Research, "Siniša Stanković", University of Belgrade, Despota Stefana 142, 11060 Belgrade, Serbia
[5] Instituto de Investigação do Medicamento (iMed.ULisboa), Faculdade de Farmácia, Universidade de Lisboa, 1649-003 Lisboa, Portugal
[6] CERENA–Centre for Natural Resources and the Environment, Instituto Superior Técnico (IST), Universidade de Lisboa, Av. Rovisco Pais, 1049-001 Lisbon, Portugal
* Correspondence: mduarte@ff.ulisboa.pt (N.D.); patricia.rijo@ulusofona.pt (P.R.)

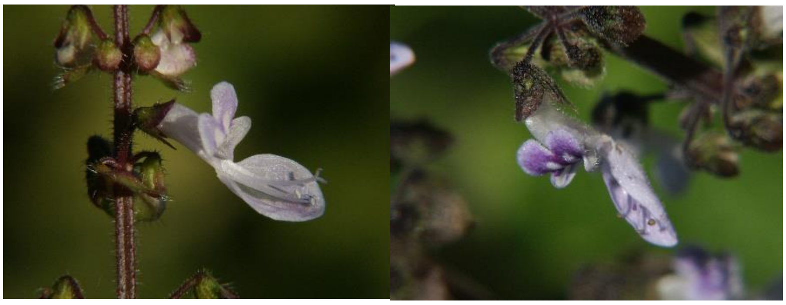

Figure S1: Selected *Plectranthus* spp. photos: *P. aliciae* (left) and *P. stylesii* (right).

Table S1. *Plectranthus spp.* voucher numbers and respective traditional medicine uses.

Plectranthus spp.	Voucher Number	Traditional medicine uses [reference]
P. aliciae	828/2007	Not described
P. amboinicus	649/2007	Digestive, skin, respiratory, infections/fever, genito-urinary, circulatory and blood, nervous, sensory, poisons treatment [2]
P. barbatus	831/2007	Digestive, skin, respiratory, infections/fever, genito-urinary, circulatory and blood, nervous, sensory, poisons treatment, cytotoxic [2,5]
P. ecklonii	832/2007	Skin [2]
P. fruticosus	C. Marques, S/N° LISC	Respiratory, poison treatment[2]
P. hadiensis	438/2010	Digestive, skin, respiratory [2]
P. hereroensis	160382	Digestive [2]
P. japonicus	1998/2009	Not described
P.madagascariensis	575/2005	Skin, Respiratory, poison [2]
***P.madagascariensis* var. "Lynne"**	LISU: 261633	Not described
P. malvinus	573/2008	Not described
P. oertendahlii	776/2008	Not described
P. reflexus	438/2010	Not described
P. stylesii	837/2007	Not described
P. strigosus	C. Marques S/Nº LISC	Not described
P. zuluensis	645/2007	Not described

Table S2: Antimicrobial activity of *Plectranthus* spp. extracts using the well diffusion method (zone of inhibition in mm).

**Extracts**	**Gram-positive bacteria**		**Gram-negative bacteria**	
	***E. faecalis* (ATCC 29212)**	***S. aureus* (ATCC 25923)**	***E. coli* (ATCC 25922)**	***P. aeruginosa* (ATCC 27853)**
P. aliciae acetonic extract	**19**	**20**	5	5
P. japonicus acetonic extract	5	**10**	5	5
P. madagascariensis 'Lynne' acetonic extract	**16**	**17**	5	5
P. stylesii acetonic extract	5	**10**	5	5
P. strigosus acetonic extract	5	**9**	5	5
Positive control	20 VAN	22 VAN	33 NOR	32 NOR
Negative control (DMSO)	5	5	5	5

Values boldly written are considered active (inhibition zone >5mm). VAN – vancomycin inoculated at 1mg/mL; NOR – norfloxacin inoculated at 1mg/mL.

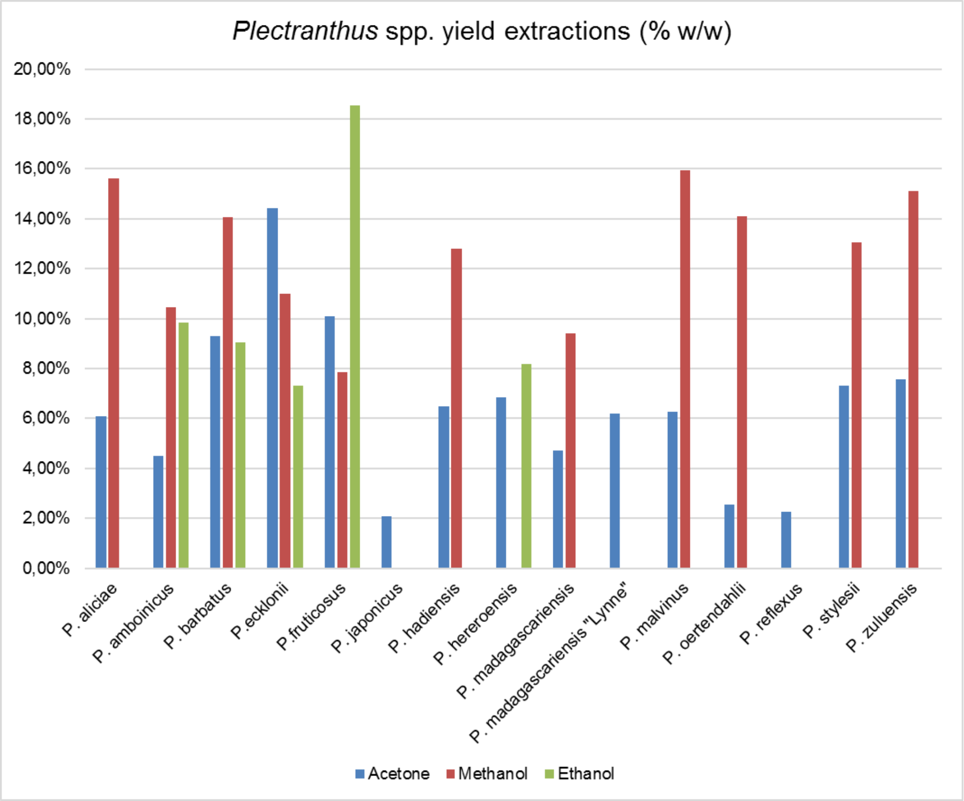

Figure S2. Percentage yield of the *Plectranthus spp.* extracts (expressed in percentage dry weight (% *w*/*w*)) according to the extracting solvents used.

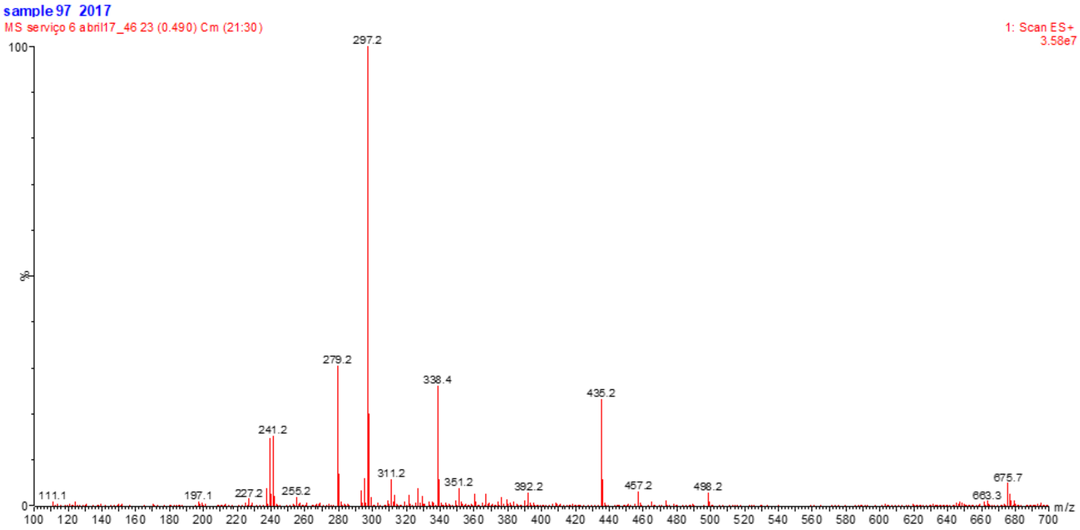

Figure S3. Parvifloron D Mass Spectroscopy spectra (MS/MS spectra).
